# Impact of curing temperature on accuracy and physical properties of additively manufactured FDPs and bar specimens

**DOI:** 10.1038/s41598-025-28886-7

**Published:** 2025-11-21

**Authors:** Julian Nold, Beatrice Arnold, Kirstin Vach, Christian Wesemann, Siegbert Witkowski, Jörg Lüchtenborg, Benedikt Christopher Spies

**Affiliations:** 1 Department of Prosthetic Dentistry, Center for Dental Medicine, Faculty of Medicine, Medical Center ‑ University of Freiburg, University of Freiburg, Hugstetterstr. 55, 79106 Freiburg, Germany; 2 Institute of Medical Biometry and Statistics, Medical Center, Faculty of Medicine, University of Freiburg, University of Freiburg, Hugstetterstr. 55, 79106 Freiburg, Germany; 3 Department of Orthodontics, Justus Liebig University Giessen, Schlangenzahl 14, 35392 Giessen, Germany

**Keywords:** Additive manufacturing, Curing temperature, Fracture strength, Stereolithography (SLA), Fixed dental prostheses, Subtractive manufacturing, Engineering, Health care, Materials science, Medical research

## Abstract

Additive manufacturing based on photopolymer resins requires additional light curing during postprocessing to achieve the final mechanical properties. The present in-vitro study aimed to evaluate the impact of the curing temperature during postprocessing on the fracture resistance and accuracy of both additively manufactured four-unit fixed dental prostheses (FDPs) and bar-shaped, ISO-conform specimens. All samples were fabricated by either additive manufacturing using two different light-curing resins or subtractive manufacturing using polymethyl methacrylate. Three different curing temperatures were evaluated (40 °C, 60 °C, 80 °C). All bars (*n* = 136) were subjected to a three-point bending test after 68 of them had been stored in water for 24 h. All bridges (*n* = 90) were analyzed for dimensional accuracy and afterwards subjected to static loading until fracture. Additively manufactured bar-shaped specimens exhibited significantly higher flexural strength compared to the subtractively manufactured counterparts. In contrast, all but one group (*p* = 0.082) of the additively manufactured four-unit FDPs demonstrated significantly lower fracture strength than the subtractively manufactured bridges (*p* < 0.024). Post-curing temperature had no significant effect on accuracy for the temporary resin (*p* > 0.469), whereas the permanent resin showed significant differences for some regions of interest and temperature combinations. For the recommended curing duration of 40 min, the lowest tested curing temperature of 40 °C already resulted in a sufficient degree of cure and mechanical properties with no general benefit to higher curing temperatures, indicating their use to be unnecessary.

**Supplementary Information:**

The online version contains supplementary material available at 10.1038/s41598-025-28886-7.

## Introduction

Additive manufacturing (AM) in dentistry has experienced continuous growth over the past decade. This has been driven by the increased accessibility and faster, higher-resolution printing offered by desktop 3D printers. The increase in material selection has opened up further areas of application. Recently, there has been a significant increase in the number of materials that are not only suitable for temporary restorations but are also approved for definitive crowns and bridges^1^.

In the field of dentistry, vat photopolymerization, whereby a liquid photopolymer in a vat is selectively cured by light-activated polymerization^2^, has become the most widespread additive manufacturing technology. Stereolithography (SLA) and digital light processing (DLP) can be considered as the predominant VAT technologies. In the case of SLA, the polymerization is achieved using a directed UV-laser spot, whereas in DLP, a whole layer is simultaneously polymerized using a UV-light mask^3^. After printing, the parts have to be cleaned of excess resin and then post-polymerized, also known as post-curing, using UV light.

There is still limited research on how different factors influence the additive manufacturing process and the resulting properties of printed parts^4–6^. Therefore, clear recommendations for specific clinical indications are still limited^6,7^.

One such parameter is the post-processing of the printed workpieces^8^. Depending on the type of additive manufacturing and the material used, post-processing is considered mandatory, especially in vat polymerization techniques that use photosensitive resin^9^. Post-processing must be performed with a high level of care, as it can have a significant impact on the final mechanical properties and biocompatibility of the printed product^5,10^. While post-processing can be divided into several steps, post-polymerization has to be considered as essential using vat polymerization techniques, as the light exposure during the printing process is generally insufficient to ensure complete polymerization of the material^6,11^. Proper post-curing is crucial to ensure that manufacturer-specified strength values such as flexural strength and modulus of elasticity are met, and to guarantee the necessary biocompatibility—meaning no release of potentially allergenic or toxic substances^12^. Curing setting and factors such as exposure time and intensity, temperature, and the type of UV unit used can have a decisive influence on material properties^6,13,14^.

Despite its relevance, little is known about what extent the post-processing curing temperature affects the properties of complex morphologies such as FDPs.

Therefore, this study aimed to investigate four objectives: (1) to compare the flexural strengths and fracture strength of subtractively manufactured versus additively manufactured reference bodies subjected to different curing temperatures according to ISO standards; (2), evaluate the impact of 24 h of water storage on fracture strength and flexural strength of forementioned specimens; (3) to evaluate the dimensional accuracy of four-unit FDPs cured at different temperatures; (4) to analyze the effect of post-curing temperatures on fracture strength of four-unit FDPs. The null hypothesis assumed that the manufacturing method, the curing temperature, and the water storage did not influence the flexural and fracture strength, as well as the dimensional accuracy.

## Materials and methods

### Fabrication of bar-shaped specimens

**Fig. 1:**
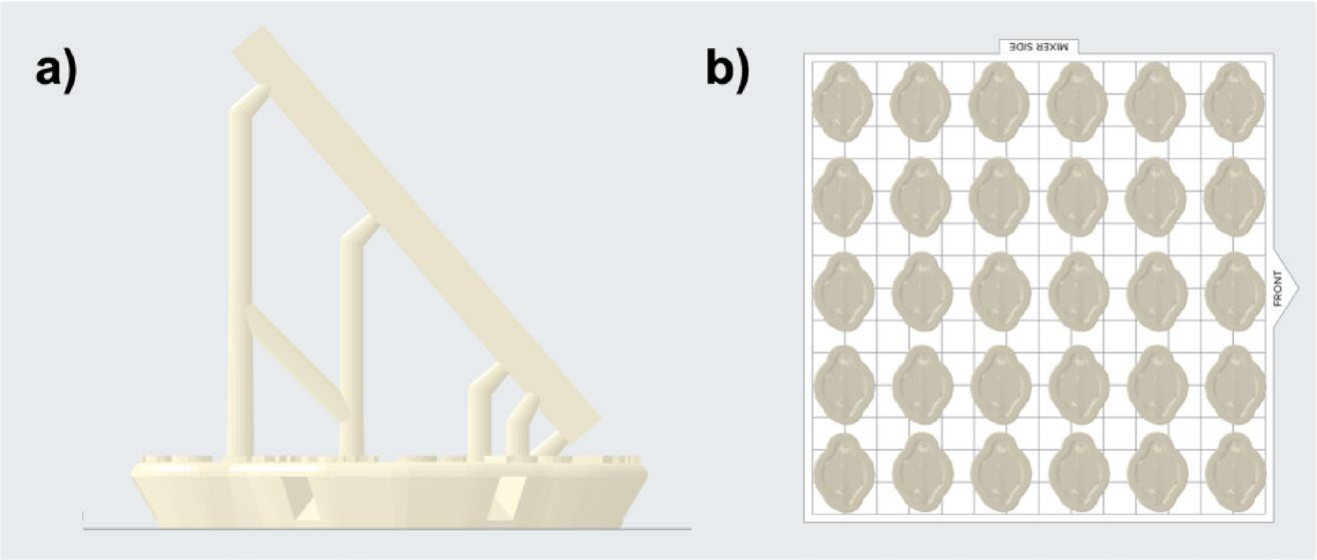
Illustration of the bar-shaped specimens and **a**) their printing orientation, including their support structures and raft, as well as **b**) their placement on the print platform.

A bar-shaped specimen with dimensions of 25 × 2 × 2 mm was designed using CAD software (Tinkercad, 2020, Autodesk, San Rafael, USA^15^, and exported as a standard tessellation language (STL) file. Acting as the control group, 16 bar specimens were subtractively manufactured out of polymethyl methacrylate (PMMA) blanks commonly used for provisional restorations (Multilayer PMMA Disc, A3, LOT: 67974, Expiration Date: 2027-05-01, Dentsply Sirona, Charlotte, NC, USA). This was achieved using a five-axis milling machine (MC X5, Dentsply Sirona) equipped with the recommended PMMA bur set (0.5 mm, 1.0 mm, and 2.5 mm bur, Dentsply Sirona) and the highest quality setting (inLab CAD, 20.0.1, Dentsply Sirona^16^,. For additive manufacturing of the bar-shaped specimens, the design was placed in a diagonal orientation on the print platform (PreForm Software, 3.9.0, Formlabs, Somerville, MA, USA^17^,. 138 specimens were printed using two hybrid ceramic resins for temporary bridges (Temporary CB, A3, LOT: 600130, expiration date: 2022-08-07, Formlabs; *n* = 69) and permanent crowns (Permanent C, A3, LOT: 600164, expiration date: 2022-09-25, Formlabs; *n* = 69) using an SLA printer (Form 3, Formlabs) using a layer height of 50 μm (Fig. 1).

Postprocessing of the printed samples included a 10-minute wash in 99% isopropanol (Form Wash, Formlabs) followed by UV-curing for 20 min at either 40 °C, 60 °C, or 80 °C (Form Cure, Formlabs). Thereafter, the raft and supports were removed, and the bars were sandblasted using 50 μm glass beads (Perlablast, BEGO, Bremen, Germany) at 1.5 bar. This was followed by a repeat of the prior 20-minute UV-curing utilizing the previously used temperature settings.

**Fig. 2:**
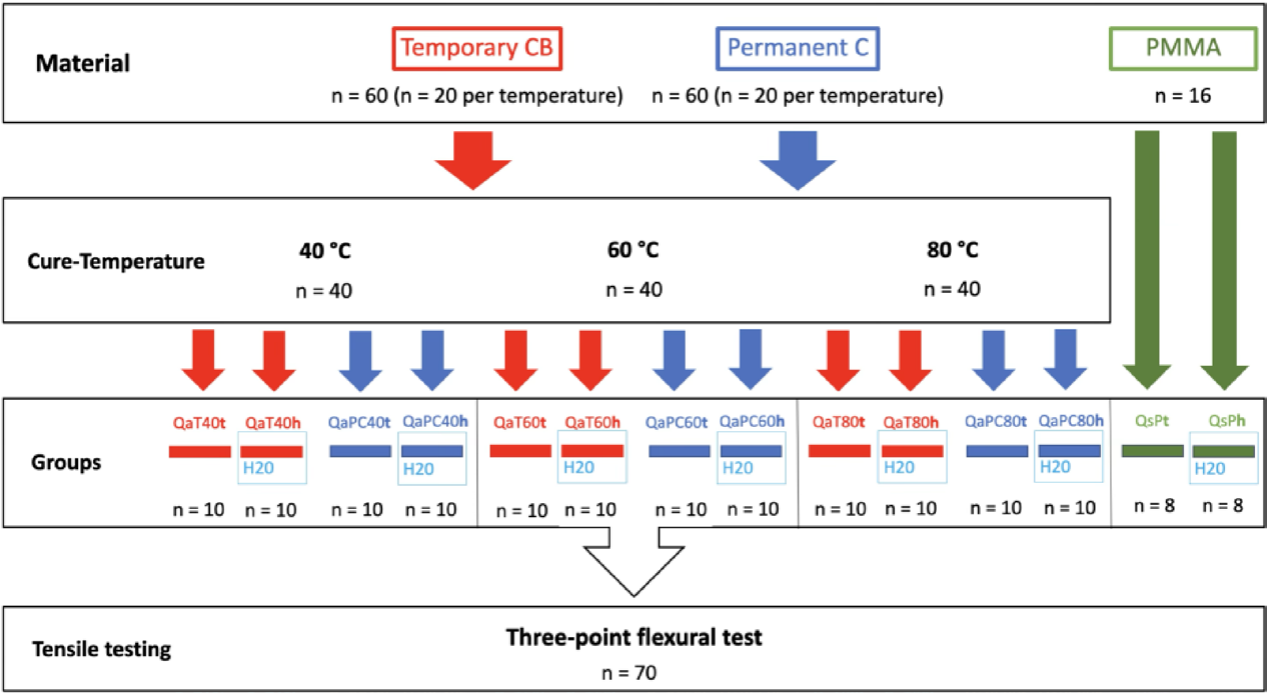
Schematic experimental setup of the bar-shaped specimens (Q = bar-shaped test specimens, n = number of specimens, a = additive, s = subtractive, T = temporary CB, PC = permanent crown, P = PMMA, t = dry storage, h = water storage, 40/60/80 = post-processing temperature: 40 °C, 60 °C, 80 °C).

After completion of postprocessing, all specimens were measured in height and width using a digital caliper with an accuracy of 0.01 μm (DealMux, Guangzhou, China). Subsequently, ten specimens from each material and curing temperature group were immersed in distilled water and stored at 37 °C for 24 h. As a control, an equal number of specimens was stored under identical conditions at 37 °C for 24 h except in a dry condition (Fig. 2).

### Raman spectroscopy & Vickers hardness testing

**Fig. 3:**
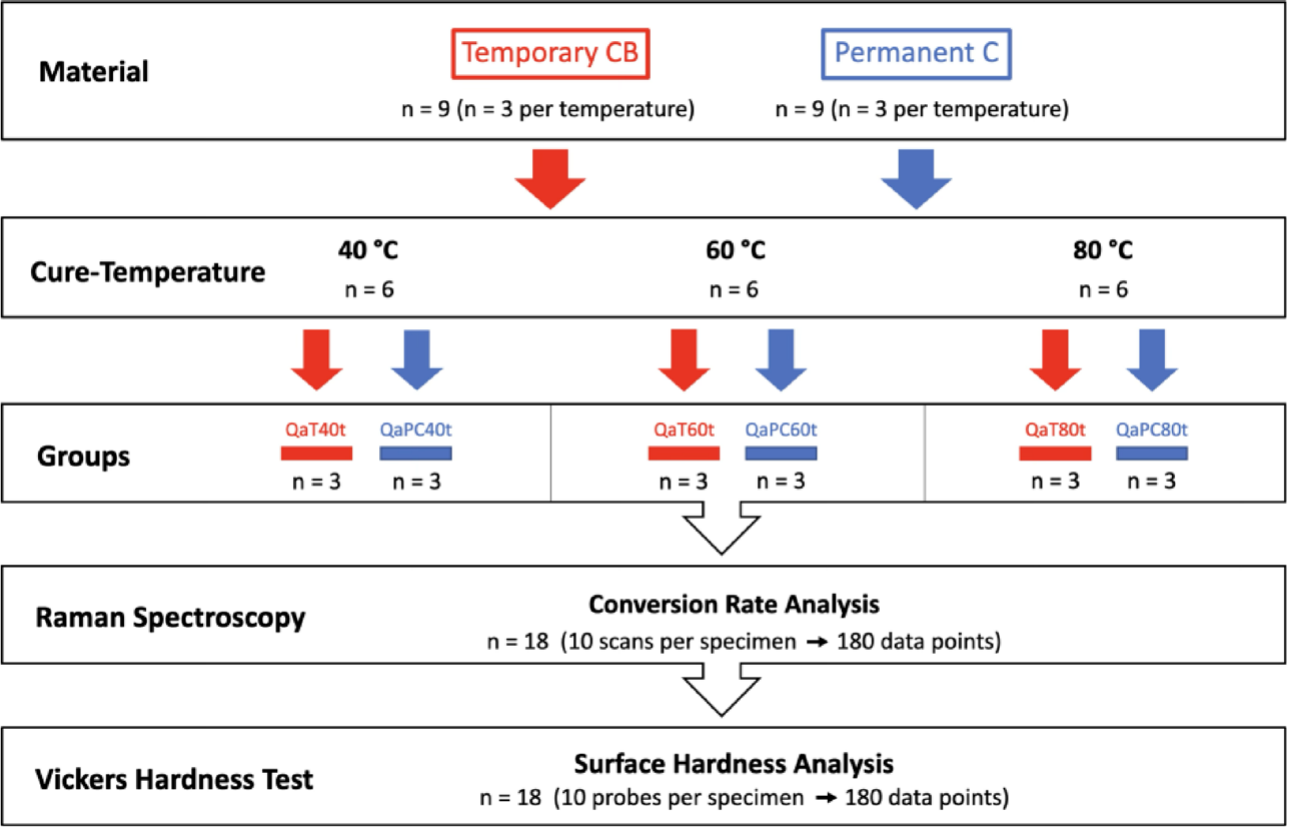
Schematic experimental setup of the Raman spectroscopy and Vickers hardness testing with bar-shaped specimens (Q = bar-shaped test specimens, n = number of specimens, a = additive, s = subtractive, T = temporary CB, PC = permanent crown, P = PMMA, t = dry storage, h = water storage, 40/60/80 = post-processing temperature: 40 °C, 60 °C, 80 °C).

Additionally, three specimens per material and curing temperature (*n* = 18) were analyzed using Raman Spectroscopy and subjected to Vickers Hardness testing (Fig. 3). Ten data points were acquired per specimen for both Raman spectroscopy and hardness measurements.

To determine the degree of conversion (DC), Raman measurements were performed using a 785 nm laser at 100% power and an integration time of 10 s, with each measurement repeated three times. Spectra were recorded for each of the resins and post-cured samples in the wavelength range of 1400 cm⁻¹ to 1800 cm⁻¹. The DC calculation was performed by comparing the relative change of the band at 1637 cm⁻¹, which represents the C = C stretching mode, with the aromatic C = C band at 1609 cm⁻¹ reference band before and after polymerization.

The degree of conversion was calculated using the following Eq. (1):

1 $$\:\text{D}\text{C}\:\left[\text{\%}\right]=100\text{*}\left(1-\frac{{R}_{cured}}{{R}_{Resin}}\right);R=\:\frac{{I}_{1637}}{{I}_{1609}}$$

Vickers hardness (HV) testing was conducted using an automated testing device (Duramin-40 AC3, Struers, Ballerup, Denmark). A Vickers indenter (square-based diamond pyramid with an angle of 136°) was applied to the specimen surface under a controlled force (HV 0.5; 500 g). The resulting indentation was measured and used to calculate the hardness value.

### Flexural property evaluation of bar-shaped specimens

The flexural strength of all three materials and the influence of the curing temperature, as well as water storage, were determined in a three-point bending test in accordance with ISO 10,477^18^.

The three-point bending test until fracture was performed using a universal testing machine (Z010/TN2S, ZwickRoell, Ulm, Germany) with a loading span of 20 mm and a crosshead speed of 1 mm/min. The maximum flexural strength, σ, in megapascal (MPa) was calculated with the following Eq. 2:

2 $$\sigma =\:\frac{{3} Fl}{{2} wh^{2}}$$

*F* is the maximum load in Newton, *l* is the distance between the supports in millimeters (20 mm), *w* is the width in millimeters (2 mm), and *h* is the height in millimeters (2 mm).

The Flexural Modulus, E, was also simultaneously calculated with the following Eq. 3:

3 $$E =\:\frac{{L}^{3} m}{{4} bh^{3}}$$

*m* is the slope of the initial straight-line portion of the load–deflection curve in N/mm, *L* is the distance between the supports in millimeters (20 mm), *b* is the width in millimeters (2 mm), and *h* is the height in millimeters (2 mm).

### Fabrication of FDPs

To fabricate the four-unit FDPs, a previously established design was used^19^. This digital FDP design (DentalCAD 2.1 Riga, exocad, Darmstadt, Germany^20^, was based on a chamfer preparation of the upper left first premolar (24, according to the FDI scheme) and second molar (27), as well as two pontics spanning the distance of the upper left second premolar (tooth 25) as well as the upper left first molar (26). The resulting cross-section of the connector between 24 and 25 measured 19.2 mm², 16.1 mm² between 25 and 26, and 22.6 mm² between 26 and 27.

**Fig. 4:**
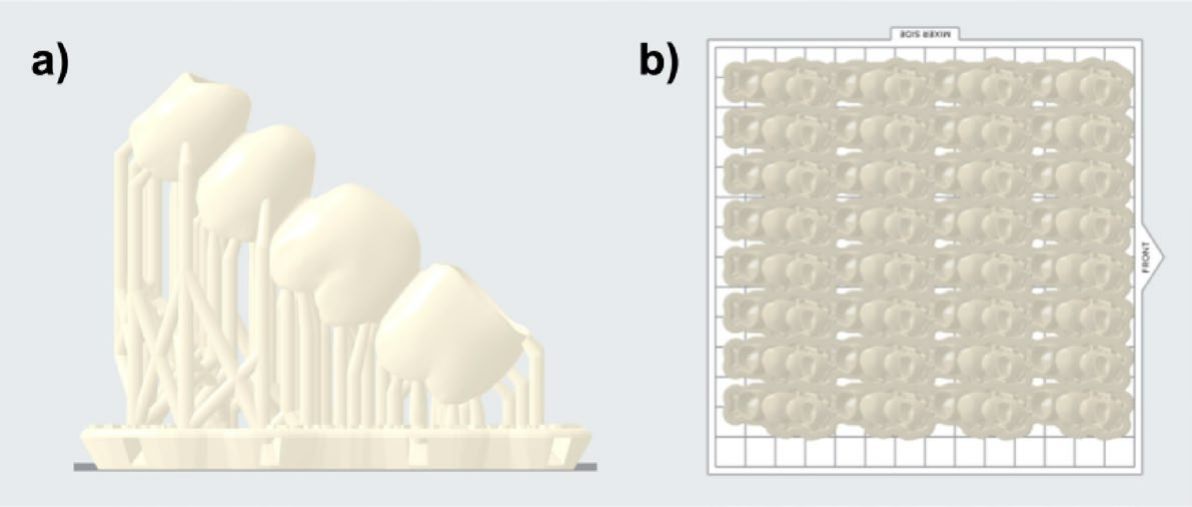
FDPs were printed utilizing an angled printing orientation: (**a**) FDP with supports and raft and (**b**) 32 FDPs nested on the build platform.

All additively manufactured FDPs (*n* = 64) were printed in a diagonal orientation (Fig. 4). Supports were placed only on the external surface; no internal supports were used. Post-processing was performed identically to that described for the bar-shaped specimens.

**Fig. 5:**
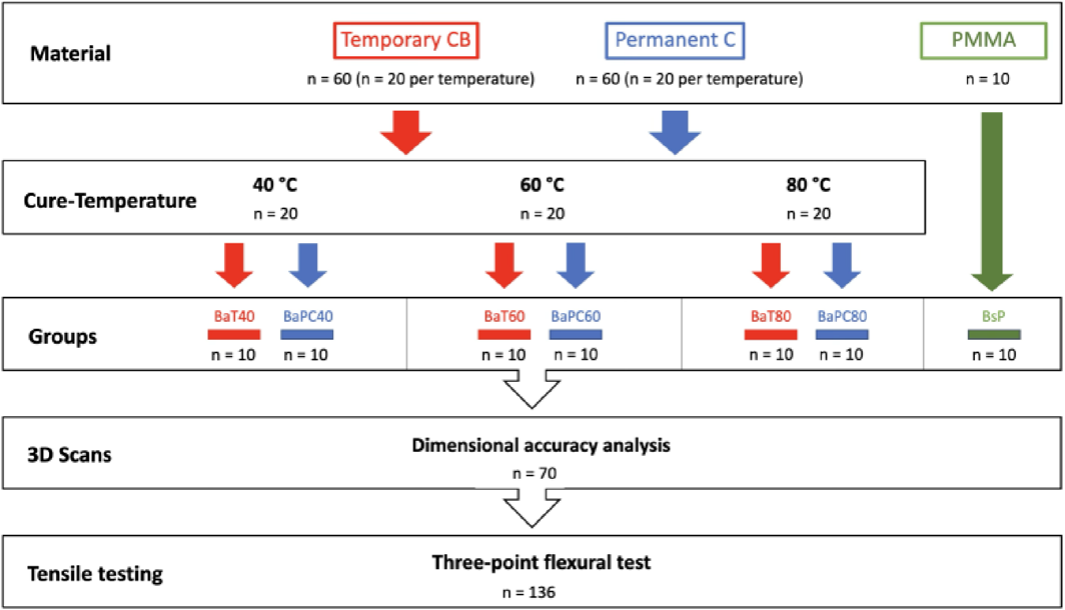
Schematic experimental setup of the four-unit bridges (B = bridge, n = number of bridges, a = additive, s = subtractive, T = temporary CB, PC = permanent crown, P = PMMA, 40/60/80 = post-processing temperature: 40 °C, 60 °C, 80 °C).

Following the same procedure as for the bar-shaped specimens, ten four-unit FDPs (BsP) were fabricated from PMMA blanks (Multilayer PMMA Disc) using a 5-axis milling machine (MC X5) to serve as the control group (Fig. 5).

### Scan and three-dimensional evaluation of FDPs

**Fig. 6:**
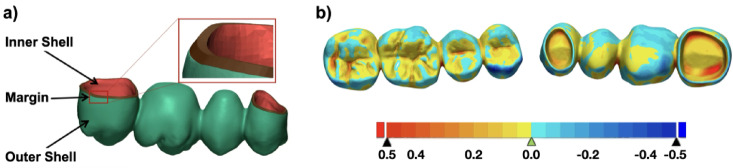
**(a)** Representation of the surface segmentation for the 3D comparison (Inner Shell = internal surface (red), Margin = preparation margin (brown), Outer Shell = external surface (green)), (**b**) representation of the best-fit alignment and heatmap of both the original STL file and the scan of an additively manufactured FDP.

After post-processing, all four-unit FDPs were digitized using an intraoral scanner (Primescan, Cerec SW 5.1.2, Dentsply Sirona^21^. Each bridge was scanned manually and extraorally, ensuring that the external and internal surfaces were completely captured. Scans were exported as an STL file and evaluated for dimensional accuracy using metrology software (Geomagic Control X, 2020.0.1, 3D Systems, Rock Hill, USA^22^,. For this, the scans of the FDPs were superimposed onto the original design, first by utilizing the initial automatic alignment option, which was visually checked, and if needed, adjusted by hand. The final alignment was achieved by using the entire surface as well as the ‘Best Fit Alignment’ algorithm by Gaus. For analyzing the dimensional accuracy, the scanned surface was segmented into three areas: external surface, internal surface, and preparation margin (Fig. 6). Dimensional deviations were quantified via residual sum of squares.

### Preparation of object holders and cementation

**Fig. 7:**
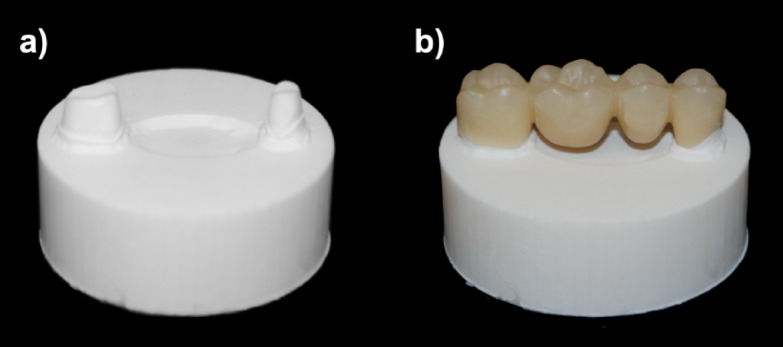
Design of the object holder with (**a**) the two prepared teeth visible and (**b**) the FDP cemented on top.

To design a standardized object holder, digitally modeled abutment teeth 14 and 17 (with a 0.6 mm chamfer preparation were exported as an STL file, including their spatial relationship (DentalCAD). Furthermore, a cylindrical base (4 cm in diameter and 1.5 cm in height) was generated (Tinkercad) and merged with the abutment teeth into a single STL file (MeshMixer, 3.5, Autodesk^23^ (Fig. 7).

Based on this design, 70 object holders were printed in acrylic resin (Rigid Resin, Formlabs) on an SLA printer (Form 3) using a layer height of 50 μm. After the print was completed, the object holders were cleaned for 15 min in 99% isopropanol (Form Wash), followed by UV-curing for 30 min at 60 °C (Form Cure).

All FDPs were luted to their object holders with zinc oxide-based cement for temporary cementation (TempBond NE, Kerr, Bioggio, Switzerland) following the recommendations of the manufacturers at a controlled application force of 80 N.

### Static loading of FDPs

**Fig. 8:**
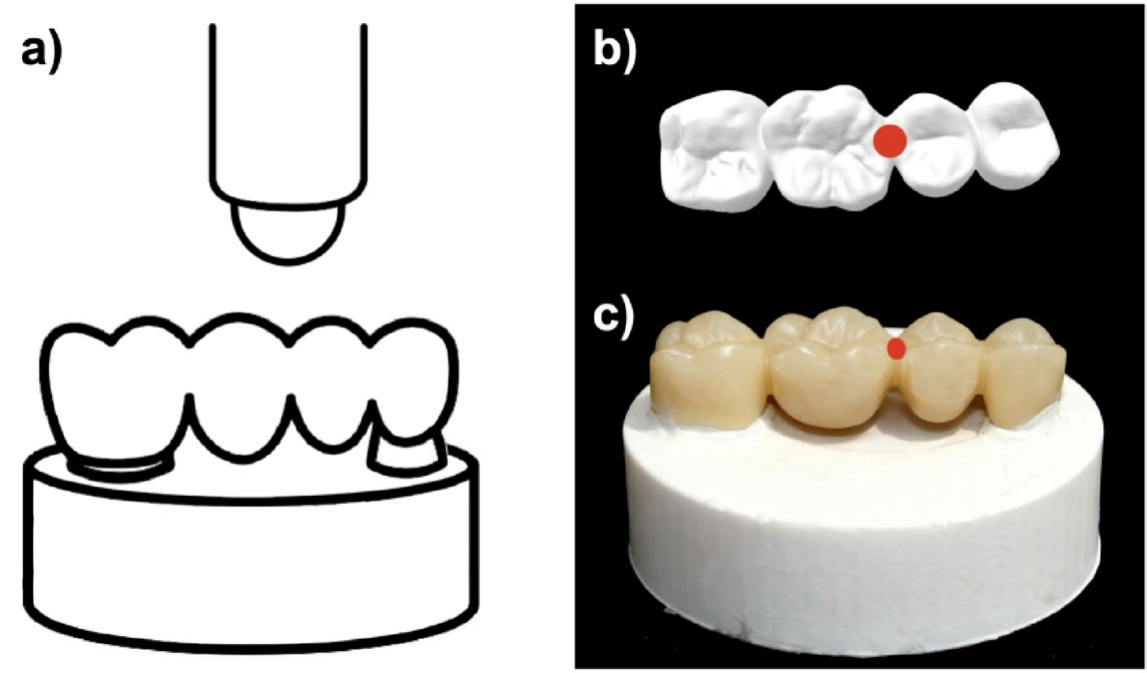
Visualization of the static fracture test with (**a**) sketch of the loading setup with the ball-shaped antagonist and b, c) highlighting (red) the contact area between the metal ball and FDP during static loading; (**b**) occlusal view of the STL, (**c**) bucco-occlusal view of one specimen cemented on the object holder.

All bridges were loaded until fracture, with the load being applied to the marginal ridge of 25 and 26 (Fig. 8) at a speed of 10 mm/min by a ball-shaped (10 mm diameter) metal antagonist. This test was performed using a universal testing machine (Z010/TN2S), and the maximum load (Fmax) was recorded.

### Statistical analysis

A sample size calculation was performed to demonstrate differences between the various hypotheses with a power of 90%. It revealed that 7 specimens per group are required to show a flexural strength difference of 15 MPa, as well as a 0.02 difference in dimensional accuracy (RMS) with a power of 90% between two groups. To show group differences between the additive materials of 500 MPa in flexural modulus and 5% in degree of conversion, 10 measurements are necessary.

For the descriptive statistical analysis of the data, the mean, median, standard deviation, minimum, and maximum values were calculated. Boxplots were generated to provide a graphical representation of the data. To evaluate the effect of water storage on fracture and flexural strength within each group, as well as the influence of curing temperature and material choice independently of water storage on dimensional accuracy, fracture strength, degree of conversion, and hardness, mixed linear models were employed. For subsequent pairwise comparisons, Scheffé’s correction was applied to account for multiple testing. For all further subgroup comparisons, non-parametric statistical methods were used due to the skewness of the distribution. When comparing two groups, the Wilcoxon test was applied; for multiple groups, the Kruskal-Wallis test was initially conducted. Post hoc pairwise comparisons were performed using Dunn’s test with Holm correction to adjust for multiple testing. The significance level was set at 5%. All statistical analyses were performed using STATA 17.0 (StataCorp, College Station, Texas, USA).

## Results

### Flexural property evaluation of bar-shaped specimens

**Fig. 9:**
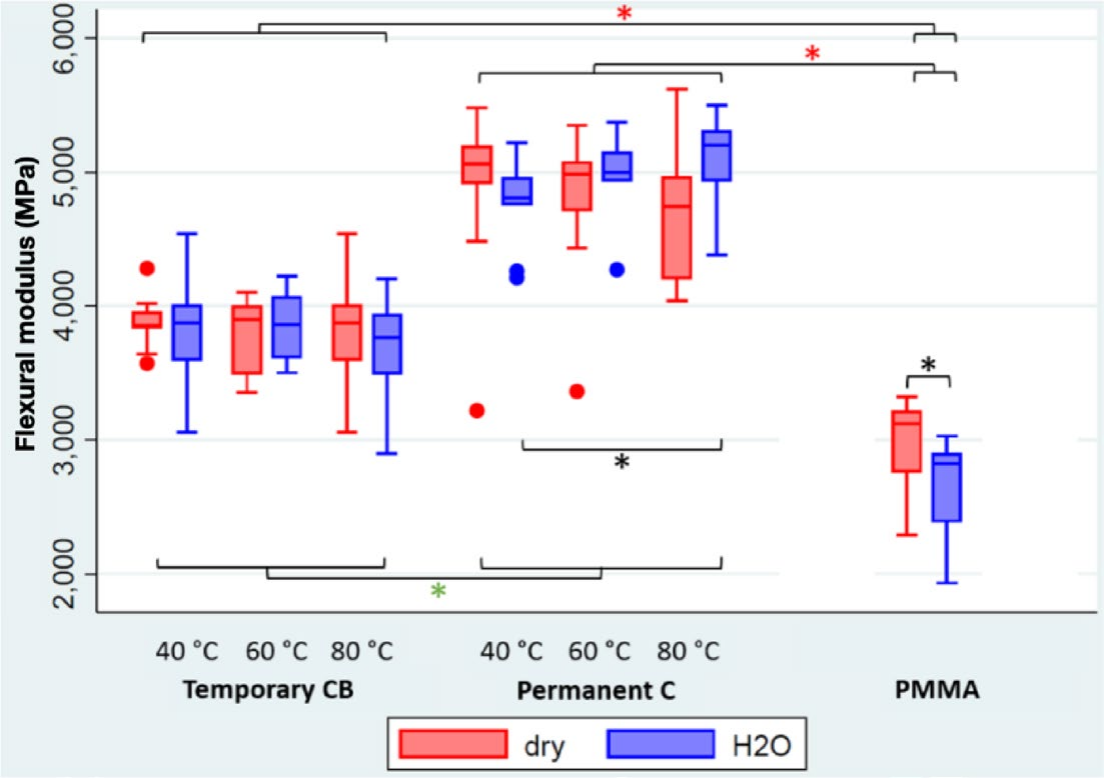
The temperature values indicate the respective post-polymerization temperature of the materials: Temporary CB (additive), Permanent C (additive), and PMMA (subtractive). “Dry” (red) denotes samples stored in dry conditions, and “H₂O” (blue) denotes samples stored in water. The black asterisks indicate significant differences in flexural modulus values within the same material group (PMMA: Wilcoxon ranksum test,.

The additively manufactured groups QaPC achieved the highest mean values for flexural strength (mean: 151.4–159.7 MPa), regardless of whether they were stored dry or immersed in water for 24 h beforehand (Table 1; Fig. 9). All six groups of QaT (mean: 134.9–141.1 MPa) revealed lower results compared to groups QaPC, while the milled groups QsP showed the overall lowest values (mean: 106.16–116.26 MPa). Neither curing temperature nor storage conditions showed a statistically significant effect within each material group (curing temperature: *p* > 0.05; storage condition: *p* > 0.05).

**Table 1:** Mean and standard deviation (SD) of the flexural strength of the additive and subtractive manufactured bar-shaped specimens in megapascals (MPa). p(40°), p(60°) and p(80°) lists the p-values for the comparison within the same material (linear mixed models), while p(QaPC), p(QaT) and p(QsP) compares each group to the corresponding group of each material (dunn test with Holm correction) and p(t) relates to a dry environment while p(h) corresponds to 24 h of storage in water (linear mixed models). Statistically significant values (*p* < 0.05) were marked with an asterisk.

Group	*N*	Mean	SD	*p*(40°)	*p*(60°)	*p*(80°)	*p*(t/h)	*p*(QaT)	*p*(QaPC)	*p*(QsP)
QaT40t	10	137,3	10,4	/	0,987	0,924	0,643	/	**0**,003*	**0**,043*
QaT40h	10	134,9	14,5	/	0,925	0,447		/	**0**,032*	**0**,016*
QaT60t	10	136,3	15,1	0,987	/	0,973	0,516	/	**0**,041*	**0**,032*
QaT60h	10	136,8	08,2	0,925	/	0,674		/	**0**,013*	**0**,009*
QaT80t	10	134,9	14,5	0,924	0,973	/	0,255	/	**0**,032*	0,061
QaT80h	10	141,1	8,3	0,447	0,674	/		/	0,079	**0**,005*
QaPC40t	10	159,7	7,9	/	0,763	0,903	0,084	**0**,003*	/	**< 0**,001*
QaPC40h	10	151,4	8,7	/	0,477	0,943		**0**,032*	/	**< 0**,001*
QaPC60t	10	154,0	16,9	0,763	/	0,960	0,868	**0**,041*	/	**< 0**,001*
QaPC60h	10	157,7	9,7	0,477	/	0,693		**0**,013*	/	**< 0**,001*
QaPC80t	10	156,2	25,5	0,903	0,960	/	0,343	**0**,032*	/	**< 0**,001*
QaPC80h	10	153,3	16,4	0,934	0,693	/		0,079	/	**< 0**,001*
QsPt	8	115,3	18,2	/	/	/	0,123	**0**,032*	**< 0**,001*	/
QsPh	8	106,2	22,1	/	/	/		**0**,005*	**< 0**,001*	/

Permanent C: dunn test with holm correction). The green asterisks indicate significant differences between the results of the two additive groups (dunn test with holm correction). The red asterisks indicate significant differences between the results of the additive groups and the subtractive comparison group (dunn test with holm correction).

### Raman spectroscopy & Vickers hardness testing

**Fig. 10:**
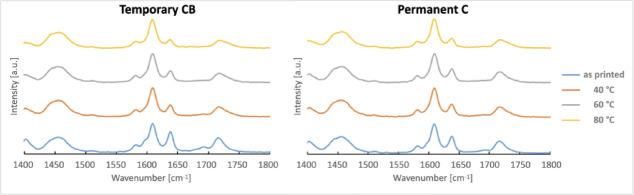
Normalized Raman spectrum of both resins, uncured as well as postprocessed, utilizing different curing temperatures (40 °C, 60 °C, 80 °C).

Figure 10 depicts the Raman spectra in the range of 1400 to 1800 cm-1. The relative change in the band at 1637 cm⁻¹, which represents the C = C stretching mode, is evident when compared with the aromatic C = C band at 1609 cm⁻¹ (reference band) for the as-printed sample and for samples cured at 40 °C, 60 °C, and 80 °C.

**Fig. 11:**
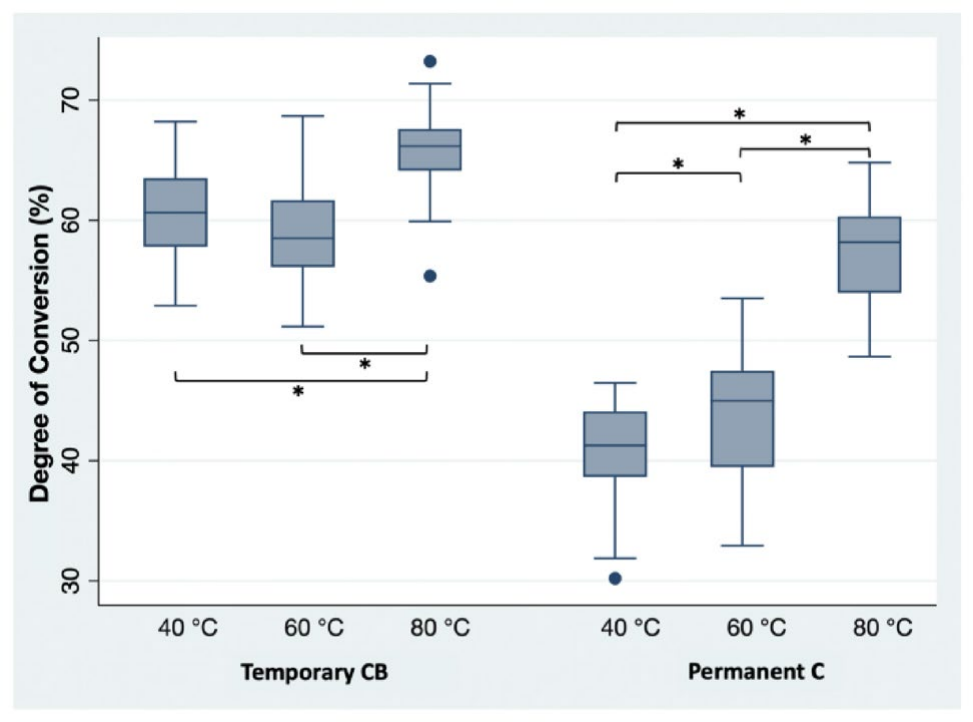
Degree of conversion (%) of the bar-shaped specimens by Raman Spectroscopy. Statistically significant values (*p* < 0.05, linear mixed models) were marked with an asterisk (*).

Calculation of the DC showed that curing temperature had a significant impact on all groups printed using the resin for permanent restorations (Permanent C), with higher curing temperature resulting in increased DC values (*p* < 0.05). For groups printed using the resin intended for temporary restorations (Temporary CB), significant differences in DC were observed (*p* < 0.05), except between the group cured at 60 °C, which did not differ significantly (*p* > 0.05) from the group cured at 40 °C (Fig. 11).

**Fig. 12:**
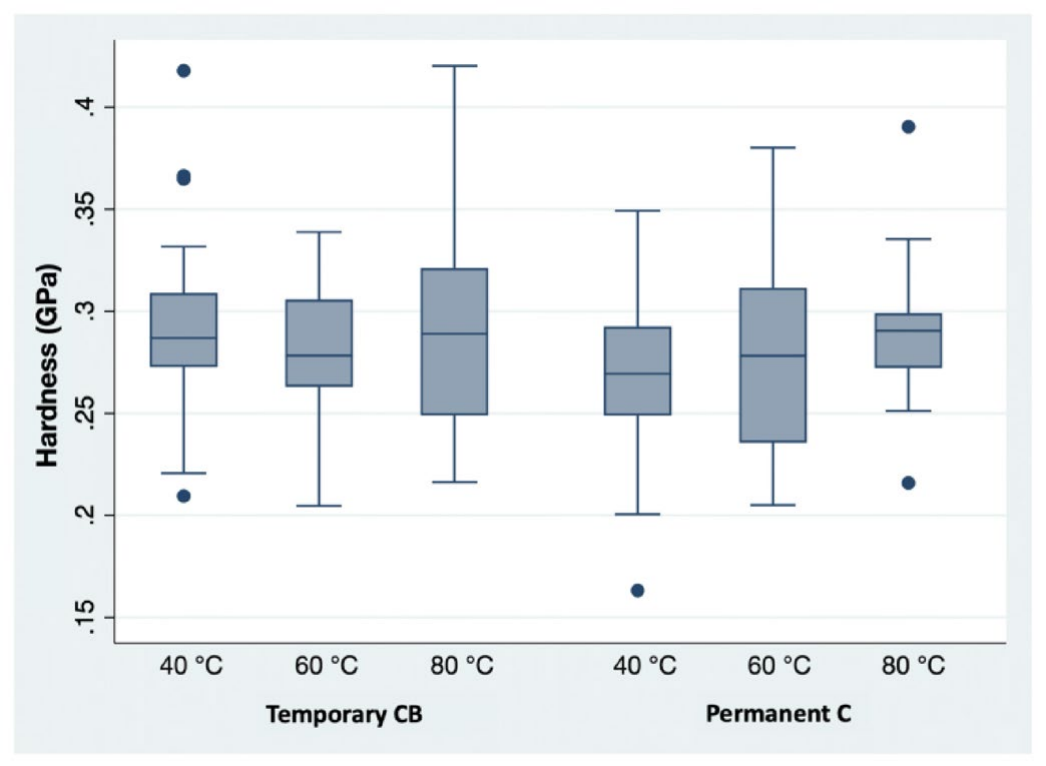
Hardness in GPa (0.5 VH) of the bar-shaped specimens.

The Vickers hardness test (Fig. 12) showed no significant difference between the curing temperatures for either the temporary material (*p* > 0.05) or the permanent material (*p* > 0.05).

### Dimensional accuracy comparison of FDPs

The dimensional accuracy analysis of the four-unit FDPs revealed that, for the outer surface, BsP exhibited the lowest mean RMS value (x̄ = 0.039), indicating the highest accuracy, followed by BaT60 (x̄ = 0.073) and BaT80 (x̄ = 0.074). BaPC80 (x̄ = 0.086) ranked mid-range, whereas BaT40 (x̄ = 0.098), BaPC40 (x̄ = 0.118), and BaPC60 (x̄ = 0.119) showed the greatest deviations from the original scan file.

On the inner surface, BsP again achieved the highest accuracy (x̄ = 0.049). BaT80 (x̄ = 0.138), BaPC80 (x̄ = 0.142), BaT60 (x̄ = 0.144), and BaT40 (x̄ = 0.171) formed the mid-range, whereas BaPC40 (x̄ = 0.184) and BaPC60 (x̄ = 0.196) exhibited the highest RMS values, indicating the lowest accuracy.

For the preparation margin, BsP demonstrated the closest agreement with the original STL file (x̄ = 0.026). BaPC60 (x̄ = 0.107) and BaT40 (x̄ = 0.128) showed the largest deviation. The RMS values of BaPC80 (x̄ = 0.078), BaT60 (x̄ = 0.091), BaPC40 (x̄ = 0.097), and BaT80 (x̄ = 0.096) fell between these extremes and can therefore be considered mid-range performers.

The overall RMS, including the entire surface of the FDP, was highest for BaPC60 (x̄ = 0.137), followed by BaPC40 (x̄ = 0.135) and BaT40 (x̄ = 0.117). The mid-range consisted of BaPC80 (x̄ = 0.099), BaT60 (x̄ = 0.091), and BaT80 (x̄ = 0.090), while BsP (x̄ = 0.041) outperformed all other groups.

**Fig. 13:**
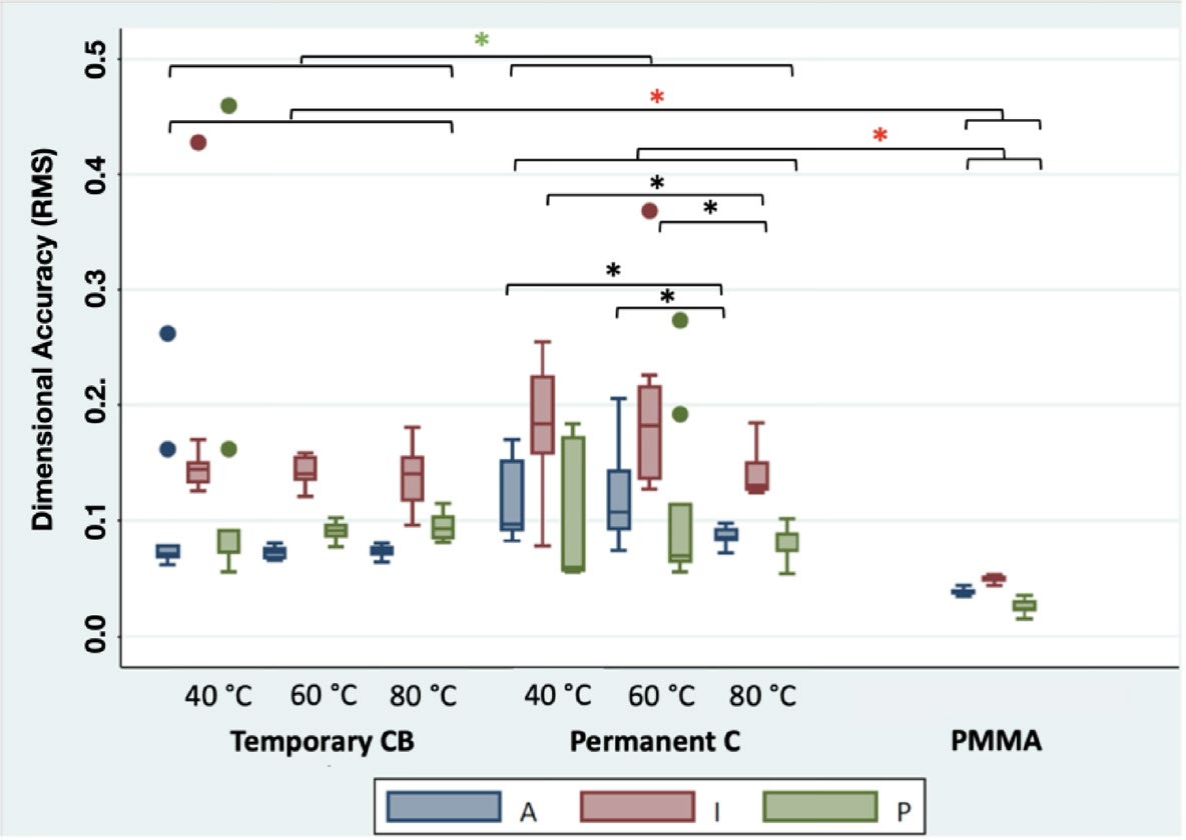
presents the results as boxplots, while the median, mean, standard deviation, and p-values for all groups across the outer and inner surfaces, as well as the preparation margin, can be found in the supplements.

Figure 13 Dimensional accuracy (RMS) split into area of interest (O: outer surface; I: inner surface; P: preparation margin) as well as material and protocol. The black asterisks indicate significant differences within the same material group. The green asterisks indicate significant differences between the results of the two additive groups. The red asterisks indicate significant differences between the results of the additive groups and the subtractive comparison group (dunn test with holm correction).

### Static loading of the FDPs

The static loading test (Table 2) of the four-unit FDPs revealed that the milled reference group BsP achieved the highest fracture force (x̄ = 366.3 N). The BaT groups demonstrated the lowest performance, with BaT40 and BaT60 showing comparable results (x̄ = 250.0 N), while BaT80 recorded the lowest mean values (x̄ = 236.9 N). Within the BaPC groups, BaPC80 attained the highest fracture force (x̄ = 254.9 N), followed by BaPC60 (x̄ = 244.8 N) and BaPC40 (x̄ = 243 N).

**Table 2:** Mean and standard deviation (SD) of the fracture load of the additive and subtractive manufactured FDPs (N), as well as the significance analysis. p(40°), p(60°), and p(80°) list the p-values for the comparison within the same material, while p(BaPC), p(BaT), and p(BsP) compare each group to the corresponding group of each material. Statistically significant values (*p* < 0.05) were marked with an asterisk (dunn test with Holm correction).

Group	Mean	SD	*p*(40°)	*p*(60°)	*p*(80°)	*p*(BaPC)	*p*(BaT)	*p*(BaP)
BaT40	250,0	62.3	/	0.550	0.315	0.444	/	0.017*
BaT60	250,0	24.5	0.550	/	0.420	0.465	/	0.017*
BaT80	236.9	60.1	0.315	0.420	/	0.205	/	0.024*
BaPC40	243,0	28.8	/	0.770	0.429	/	0.444	0.017*
BaPC60	244.8	68.7	0.770	/	0.957	/	0.465	0.021*
BaPC80	254.9	67.2	0.429	0.857	/	/	0.205	0.082
BsP	366.3	206	/	/	/	> 0.017*	> 0.017*	/

## Discussion

The primary objective of this in vitro study was to investigate the influence of different curing temperatures on the fracture strength of both additively manufactured bar-shaped specimens and four-unit FDPs. Additionally, for the bar-shaped specimens, the effect of 24 h of water storage on the flexural modulus, the degree of conversion, as well as the hardness was evaluated. For the FDPs, the dimensional accuracy of the internal, the marginal, and the outer surface was also assessed.

Accordingly, all additively manufactured bar-shaped specimens and FPDs were subjected to a post-curing temperature of either 40 °C, 60 °C, or 80 °C, with the post-curing protocol consisting of two 20-minute curing cycles.

### Manufacturing of bar-shaped specimens and FDPs

Additive manufacturing of both bar-shaped specimens (Fig. 1) as well as the FDPs (Fig. 4) was performed utilizing a diagonal printing orientation. This was chosen as a prior study, evaluating the printing orientation of additive-manufactured FDPs, revealed the highest fracture loads for the diagonally printed, non-aged samples^24^. For consistency reasons, the same orientation was chosen for the bar-shaped specimens, even though the aforementioned study revealed a vertical printing orientation for those samples as favourable.

### Flexural property evaluation of bar-shaped specimens

For all but one group (QaPC40) of the additively manufactured bar-shaped specimens, post-curing temperature and water storage had no statistically significant effect on either flexural strength or flexural modulus. However, when comparing specimens fabricated from the permanent restoration material and stored in water for 24 h, a curing temperature of 40 °C (QaPC40h, mean: 4772 MPa) and a curing temperature of 80 °C (QaPC80h; mean: 5104 MPa) revealed a significant difference (*p* = 0.011) in terms of flexural modulus.

These findings contrast with those of Bayarsaikhan et al.^13^, who reported that the flexural strength of additively manufactured specimens increased significantly with post-polymerization temperature and curing times (*p* < 0.001). Bayarsaikhan et al. observed the highest values for a post-curing temperature of 80 °C and curing times of 90 and 120 min (147.48 ± 5.82 MPa).

While in the presented study, a slight increase in fracture load can be observed with increasing curing temperature for the permanent material, whereas no such trend was detected for the temporary material. One possible explanation is that the higher fill content of the permanent material may result in the need for a longer curing time. This relationship is well documented for restorative composites^25,26^, but to date, has not been investigated for 3D printing resins. Furthermore, following the manufacturer’s post-processing recommendation, two curing cycles of 20 min each can be considered sufficient to achieve full-depth polymerization for the bar-shaped specimens evaluated in this study. The correlation between curing time and depth of cure has already been established for restorative composites^27–29^, supporting this interpretation.

Comparing the flexural strength of the different materials revealed that all but one (QaT80h) group of bar-shaped specimens fabricated out of temporary resin achieved significantly lower values (*p* < 0.05) compared to both permanent resin and PMMA specimens. The superiority of subtractively manufactured resin bar-shaped specimens is in accordance with Wechkunanukul et al.^30^, who evaluated three different manufacturing techniques and their respective materials. While they showed a mean flexural strength between 71.09 and 125.16 MPa throughout all groups, additive manufacturing was significantly lower performing compared to both other groups, while the subtractive manufacturing group also significantly outperformed the conventional manufactured group. Furthermore, Lopez. et al.^31^ revealed a positive linear correlation between the filler wt% and flexural modulus (*r* = 0.78, *p* < 0.01), as well as flexural strength (*r* = 0.46, *p* < 0.01) while evaluating bar-shaped samples manufactured utilizing different resin compositions. This is in accordance with the information provided by the manufacturer that the permanent resin has an increased filler amount compared to the temporary resin used in this study.

### Raman spectroscopy & Vickers hardness testing

Already in 1993, Shin et al. utilized Raman spectroscopy to evaluate the depth of cure of dental resins used for direct restorations^32^. This method is commonly used to evaluate differences between the curing behavior of resin-based materials^33,34^. Consistent with the findings of the static loading test, the bar-shaped specimens fabricated from the resin intended for permanent restorations exhibited a statistically significant difference in the degree of conversion (DC, *p* < 0.05).

This aligns with a study conducted by Bayarsaikhan et al., who also evaluated the influence of different curing temperatures on DC and found significant differences^13^. Their DC values ranged between 50.41% and 63.79%, whereas the present study observed a range of 40.94% to 65.3%. Although the upper limits of DC are comparable, the considerably lower minimum values observed in the present study may be attributed to differences in the printing setup, which can affect the initial DC before post-processing^35^.

In contrast to this, Vickers hardness testing revealed no significant differences in surface hardness across curing temperatures (*p* > 0.05). This further supports the assumption that depth of cure, particularly for the temporary material, is already sufficiently achieved at a curing temperature of 40 °C when following the recommended curing time of 40 min. These findings are consistent with a study by Taneva et al.^36^, which also found no significant difference in surface hardness by Vickers hardness testing for different curing temperatures while utilizing specimens in compliance with ISO 6507−1^37^.

### Dimensional accuracy comparison of FDPs

In the second part of this study, four-unit bridges were additively manufactured using the same two resins, one for temporary restorations (Temporary BC) and one for permanent restorations (Permanent Crown), as in the first part. The FDPs of each material were divided into three subgroups, which underwent post-polymerization at temperatures of 40 °C, 60 °C, or 80 °C. As a control group, subtractively manufactured four-unit bridges made of PMMA (Multilayer PMMA Disc) were used, consistent with the first part of the study.

Each bridge was digitized using an intraoral scanner to assess dimensional accuracy before being provisionally cemented onto an additively manufactured test holder. Finally, a static loading test was conducted to evaluate the fracture strength of each FDP.

The decision to split the surface of the FDP into three regions for RMS analysis was based on the higher need for accuracy for certain areas. While it can be argued that the occlusal area with its contact points is of high need for dimensional correctness, it has to be stated that internal, and especially the marginal precision, is of the highest criticality. During the design process, the dental lab software will include a spacer for luting cement in the internal area, which isn’t the case for the marginal area, resulting in the aforementioned criticality. Furthermore, an occlusal adjustment, while also including downsides as in the case of zirconia, resulting a elaborate polishing, can be considered excusable, while marginal and internal adjustments are unacceptable and can lead to improper positioning of the FDP when not spotted before cementation.

Post-polymerization temperature had no significant influence on the dimensional accuracy of the BaT groups regarding the outer and inner surfaces, as well as the preparation margin (*p* > 0.05). However, significant differences were found for both the outer surface in the BaPC groups between BaPC40 and BaPC80 (*p* = 0.021), and between BaPC60 and BaPC80 (*p* = 0.024), as well as for the inner surface between BaPC40 and BaPC80 (*p* = 0.024) and between BaPC60 and BaPC80 (*p* = 0.017).

Katheng et al.^38^ investigate the influence of three post-polymerization temperatures (40 °C, 60 °C, and 80 °C) and durations (15 and 30 min) on the dimensional accuracy and degree of polymerization of a denture design using a simplified edentulous maxilla model. The lowest RMS value was found in the group cured for 30 min at 40 °C (0.07 ± 0.02), which differed significantly from the groups with 30 min at 60 °C (0.09 ± 0.02), 15 min at 80 °C (0.10 ± 0.01), and 30 min at 80 °C (0.11 ± 0.02) (*p* < 0.05). The highest RMS value was observed in the group with 30 min at 80 °C. These findings only partially align with the current study.

In the present study, the lowest RMS values for the outer surface were observed in the groups BaPC80 (0.083) and BaT40 (0.069), indicating a correlation between increased curing temperature and improved dimensional accuracy. For the inner surface, the groups BaPC80 (0.131), BaT60 (0.141), and BaT80 (0.141) showed the highest accuracy. In contrast, for the preparation margin, the groups BaPC40 (0.060), BaT40 (0.092), and BaT60 (0.092) yielded the best results. These findings suggest that the highest post-polymerization temperature did not consistently result in the lowest RMS values or minimal deviation from the STL reference file. The overall RMS analysis of the different materials revealed that only group BaPC80 (0.099, *p* > 0.05) showed no significant difference from group BaT80 (0.090), while all other groups significantly differed. An increase in curing temperature only improved the overall RMS for the permanent resin, resulting in a significant difference between group BaPC80 to both BaPC40 (*p* = 0.008) and BaPC60 (*p* = 0.013), while the temporary resin was unaffected in terms of RMS.

Katheng et al. acknowledged that the simplified geometry of their specimens can be seen as a limitation of their study. Differences in morphology, material thickness, and layer orientation may explain the inconsistencies observed between their results and the present study, for both the denture base and FDP.

Unkovskiy et al.^39^ also investigated the influence of post-polymerization on the dimensional accuracy of printed specimens (5 × 5 × 30 mm). In their study, 40 specimens were printed, of which 30 were post-polymerized in three different light-curing units (same wavelength: 350–520 nm) for 15 min, while 10 specimens served as a control group and were evaluated as printed. Unkovskiy et al. found that the choice of light-curing unit did not influence the dimensional accuracy of their specimens (± 0.1 mm), which is partially in accordance with the findings of this study. However, dimensional accuracy in their study was measured using a digital micrometer (0.01 mm accuracy), rather than RMS deviation as in the current work. Additionally, the curing temperature, duration, and wavelength were not varied, which the authors themselves noted as a limitation.

Herpel et al. concluded in their comparative study of milled and 3D-printed try-in dentures that post-processing steps are time- and technique-sensitive and may significantly affect the dimensional accuracy of printed parts^40^. In contrast, subtractive manufacturing revealed greater standardization and reproducibility, with post-processing having minimal influence. This finding supports the results of the present study, where the subtractive group showed significantly smaller deviations across all surface areas (preparation margin, inner, and outer surfaces) than the additive groups (*p* < 0.001), and exhibited lower and more consistent standard deviations (SD additive: outer surface 0.005–0.034; inner surface 0.013–0.091; margin 0.007–0.069/SD subtractive: outer surface 0.003; inner surface 0.003; margin 0.006).

Nevertheless, the dimensional deviations of the additively manufactured FDPs remained within a clinically acceptable range according to ISO 5725−1^41^, with measured differences from the STL reference model remaining below 250 μm.

### Static loading of the FDPs

In this study, post-polymerization temperature had no significant effect on the fracture strength of additively manufactured four-unit FDPs (*p* > 0.05). The mean fracture strength values ranged from 236.9 N to 254.9 N, whereas subtractively manufactured bridges performed significantly better, with a mean fracture strength of 366.3 N (*p* < 0.05). To the author’s knowledge, no comparable studies have investigated the influence of post-polymerization temperature on the fracture strength of additively manufactured FDPs.

Numerous studies in the literature have examined the impact of various post-processing parameters on additively manufactured single crowns. However, these findings are not directly transferable to the present study, as FDPs distribute occlusal forces differently due to their geometry, including connector size and design^42^.

Schulz et al.^43^ evaluated two different resins by comparing the fracture load of printed short-span implant-supported bridges. While the mean fracture loads (311.80 ± 96,77 N and 306 ± 94.68 N) achieved by their FDPs fall between the values obtained in this study for both printed and milled FDPs, a direct comparison is not feasible. Their FDPs featured a significantly shorter span, replacing only one premolar, and were screw-retained and implant-supported, whereas the FDPs in the present study were temporarily cemented onto test dies.

Furthermore, the subtractively manufactured control group of the present study outperformed all additively manufactured groups in terms of fracture strength. This finding is in accordance with Corbani et al.^44^, who compared milled, resin-printed, as well as metal-printed and ceramic-veneered FDPs regarding fracture strength. Focusing on their comparison of both the resin printed (Irix Max, DWS, Italy) and fully anatomically milled resin polymer (Ambarino, Creamed, Germany; Trilor, Bioloren, Italy) FDPs, the latter also achieved significantly higher fracture loads (mean: 1312 N, SD: 64 N Iris Max; mean: 1360 N, SD: 148 N, Ambarino) after thermocycling and dynamic loading. Zimmermann et al.^45^ evaluated the load-bearing capacity of FDPs fabricated using different materials and both subtractive and additive manufacturing. The best-performing polymer groups, achieving a mean fracture load of 1495 N (SD: 215 N; BRILLIANT Crios, COLTENE/Whaledent AG, Altstätten, Switzerland) and 1221 N (SD: 199 N; Telio CAD, Ivoclar Vivadent AG, Schaan, Liechtenstein), were both milled from industrially manufactured blanks, whereas the only 3D printed group (ELS Even Stronger (3D); Saremco Dental AG; Rebstein, Switzerland) exhibited the lowest fracture load (929 N, SD: 194 N). These results align with the findings of the present study and support the hypothesis that industrially manufactured polymer blanks are superior to chairside- or labside-printed polymers. This can be attributed to both the possibility for the use of higher viscosity resins during blank production, which allows for greater incorporation of inorganic fillers, and to the more controlled polymerization of the material itself.

### Combined observations

The combined testing of bar-shaped specimens and FDPs in this study was intended to evaluate whether the results obtained from standardized, ISO-conform specimens can be transferred to complex, anatomically shaped restorations. While the subtractive manufactured bar-shaped specimens demonstrated lower values for flexural strength and flexural modulus compared to all additively manufactured groups, the subtractive manufactured bridges exhibited higher fracture strength than their additively manufactured counterparts. This discrepancy highlights the importance of including anatomic designs in testing protocols to reduce deviations from clinically relevant behavior that may arise from standardized specimen geometries. Nonetheless, ISO-conform testing remains valuable for standardizing experimental setups and facilitating comparability across different studies.

A key assumption of this study was that increasing the curing temperature would enhance the curing process, based on the reaction rate–temperature relationship described by the van’t Hoff rule (also known as the RGT rule). Originally proposed in 1884 by the Dutch chemist Jacobus Henricus van ’t Hoff, this rule states that a temperature increase of 10 °C approximately doubles to triple the reaction rate^46^. A higher temperature is thus expected to accelerate polymerization by increasing the diffusion and reactivity of free radicals throughout the material^47^.

Bayarsaikhan et al. demonstrated that higher post-polymerization temperatures improved mechanical properties, although their study focused exclusively on bar-shaped specimens^13^. These findings are in contrast to those of the present study. Notably, Bayarsaikhan et al. tested a broader range of curing times, whereas the current study applied a fixed post-curing duration of 40 min, divided into two equal cycles, across all curing temperature groups. They further observed that most changes in degree of conversion (DOC) occurred at shorter curing times, and that no significant improvements were found after 60 min of post-curing. This supports the aforementioned interpretation that the manufacturer-recommended post-curing duration of 40 min was already sufficient to achieve a high degree of conversion, thus minimizing the impact of increased curing temperature.

When evaluating the limitations of this study, multiple factors can be considered. The lowest curing temperature chosen in this study, 40 °C, could still be seen as too high. It was selected because 80 °C was the maximum temperature supported by the light oven included in the Formlabs ecosystem, which is 20 °C higher than the manufacturer’s recommended curing temperature of 60 °C. The authors considered it desirable to maintain the same 20 °C difference below the recommended temperature for the lower setting. Furthermore, a curing duration of two cycles (20 min each), while based on the manufacturer’s recommendation, could also be considered too long to fully evaluate the effect of different curing temperatures.

Specimen preparation, following the manufacturer’s recommendations, included removal of support structures and glass-bead blasting, which can both cause surface loss. To minimize this, the supports were placed on easily reachable places, primarily on the occlusal surface. Furthermore, the recommended air pressure of 1.5 bar and distance to the object were tightly controlled to further minimize impact.

Although multiple parameters, such as accuracy, flexural strength, fracture strength, flexural modulus, degree of conversion, surface hardness, and the effect of 24 h of water storage, were evaluated, no mechanical aging was performed prior to testing. This is particularly noteworthy, as the additively manufactured samples showed a significantly higher flexural modulus compared to the subtractive specimens, which could lead to greater susceptibility to degradation during mechanical aging. This was already shown for three-unit FDPs in a prior study using a different resin (Denture Teeth, Formlabs), but comparison was performed with the same subtractive and less flexible material (Multilayer PMMA Disc) used in the presented study^24^. Further studies addressing these points could provide a more comprehensive clinical prognosis.

## Conclusions

Subtractively manufactured bar-shaped specimens showed lower flexural strength and modulus than all additively manufactured groups, regardless of water storage or curing temperature. In contrast, subtractively manufactured FDPs demonstrated higher fracture strength and greater dimensional accuracy, emphasizing the importance of testing anatomically shaped restorations to predict clinically relevant behaviour.

Post-curing temperature had no significant overall effect on the mechanical properties or dimensional accuracy of the additively manufactured samples. A slight improvement was observed for the permanent resin at higher temperatures, but this was only significant in one case. The temporary resin showed no consistent trend. Therefore, it could be stated that a curing temperature above 40 °C might not benefit the clinical results for the tested materials.

## Supplementary Information

Below is the link to the electronic supplementary material.

Supplementary Material 1

## Data Availability

Supporting data are provided within the manuscript, and raw data are available from the corresponding author upon reasonable request.
