# Supplementary material for: Impact of curing temperature on accuracy and physical properties of additively manufactured FDPs and bar specimens
Source: Sci Rep. 2025 Nov 21;15:41186. doi: 10.1038/s41598-025-28886-7 (PMC12638860; doi:10.1038/s41598-025-28886-7)
Supplement: Supplementary file 1 — Supplementary Material 1 [file 41598_2025_28886_MOESM1_ESM.docx]

Supplement:

**Supplementary Information 1** Dimensional accuracies of groups BaPC, BaT, and BsP for the outer surface, inner surface, and preparation margin (in RMS). Statistically significant values (p < 0.05, dunn test with holm correction) were marked with an asterisk (*).

| **Group** | **p50 (RMS)** | | **Mean (RMS)** | **SD (RMS)** | **p(40°C) C** | | **p(60°C) C** | | **p(80°C) C** | | **p(BaPC)** | **p(BaT)** | **p(BsP)** |
| --- | --- | --- | --- | --- | --- | --- | --- | --- | --- | --- | --- | --- | --- |
| **Outer surface** | | | | | | | | | | | | | |
| BaT40 | | 0.069 | 0.099 | 0.021 | | / | | 0.469 | | 0.819 | 0.058 | / | **<0.001*** |
| BaT60 | | 0.073 | 0.073 | 0.005 | | 0.469 | | / | | 1.000 | **0.009*** | / | **<0.001*** |
| BaT80 | | 0.075 | 0.074 | 0.006 | | 0.819 | | 1.000 | | / | **0.030*** | / | **<0.001*** |
| BaPC40 | | 0.097 | 0.112 | 0.034 | | / | | 0.459 | | **0.021*** | / | 0.058 | **<0.001*** |
| BaPC60 | | 0.108 | 0.119 | 0.039 | | 0.459 | | / | | **0.028*** | / | **0.009*** | **<0.001*** |
| BaPC80 | | 0.083 | 0.086 | 0.009 | | **0.021*** | | **0.028*** | | / | / | **0.030*** | **<0.001*** |
| BsP | | 0.039 | 0.039 | 0.003 | | / | | / | | / | **<0.001*** | **<0.001*** | / |
| **Inner surface** | | | | | | | | | | | | | |
| BaT40 | | 0.145 | 0.171 | 0.091 | | / | | 0.469 | | 0.739 | 0.111 | / | **0.001*** |
| BaT60 | | 0.141 | 0.143 | 0.013 | | 0.469 | | / | | 0.542 | 0.102 | / | **0.001*** |
| BaT80 | | 0.141 | 0.138 | 0.025 | | 0.739 | | 0.542 | | / | 0.459 | / | **<0.001*** |
| BaPC40 | | 0.184 | 0.184 | 0.052 | | / | | 0.499 | | **0.024*** | / | 0.111 | **<0.001*** |
| BaPC60 | | 0.183 | 0.196 | 0.070 | | 0.499 | | / | | **0.017*** | / | 0.102 | **<0.001*** |
| BaPC80 | | 0.131 | 0.142 | 0.022 | | **0.024*** | | **0.017*** | | / | / | 0.459 | **<0.001*** |
| BsP | | 0.049 | 0.049 | 0.003 | | / | | / | | / | **<0.001*** | **<0.001*** | / |
| **Preparation Margin** | | | | | | | | | | | | | |
| BaT40 | | 0.092 | 0.128 | 0.120 | | / | | 0.409 | | 0.739 | 0.271 | / | **<0.001*** |
| BaT60 | | 0.092 | 0.091 | 0.007 | | 0.409 | | / | | 0.648 | 0.288 | / | **<0.001*** |
| BaT80 | | 0.093 | 0.096 | 0.013 | | 0.739 | | 0.648 | | / | **0.047*** | / | **<0.001*** |
| BaPC40 | | 0.060 | 0.097 | 0.057 | | / | | 0.646 | | 0.559 | / | 0.271 | **<0.001*** |
| BaPC60 | | 0.070 | 0.107 | 0.069 | | 0.646 | | / | | 0.419 | / | 0.288 | **<0.001*** |
| BaPC80 | | 0.074 | 0.078 | 0.015 | | 0.559 | | 0.419 | | / | / | **0.047*** | **<0.001*** |
| BsP | | 0.024 | 0.026 | 0.006 | | / | | / | | / | **0.000*** | **0.000*** | / |
